# Sodium glucose cotransporter 2 inhibitor suppresses renal injury in rats with renal congestion

**DOI:** 10.1038/s41440-023-01437-1

**Published:** 2023-09-25

**Authors:** Akari Endo, Takuo Hirose, Shigemitsu Sato, Hiroki Ito, Chika Takahashi, Risa Ishikawa, Ayaka Kamada, Ikuko Oba-Yabana, Tomoyoshi Kimura, Kazuhiro Takahashi, Takefumi Mori

**Affiliations:** 1 Division of Nephrology and Endocrinology, Faculty of Medicine, Tohoku Medical and Pharmaceutical University, Sendai, Japan; 2 Department of Endocrinology and Applied Medical Science, Tohoku University Graduate School of Medicine, Sendai, Japan; 3 Division of Integrative Renal Replacement Therapy, Faculty of Medicine, Tohoku Medical and Pharmaceutical University, Sendai, Japan

**Keywords:** Renal congestion, Renal injury, SGLT2 inhibitor, Fibrosis, Inflammation, Mitochondria.

## Abstract

Renal congestion is an issue of cardiorenal syndrome in patients with heart failure. Recent clinical and basic studies suggest a renoprotective potential of sodium–glucose cotransporter (SGLT) 2 inhibitors. However, the effect on renal congestion and its mechanism is not fully understood. Thus, we aimed to clarify the effect of SGLT inhibition in a renal congestion model. Renal congestion was induced in the left kidney of male Sprague-Dawley rats by ligation of the inferior vena cava between the renal veins. The SGLT2 inhibitor tofogliflozin or vehicle was orally administered daily from the day before IVC ligation until two days after surgery. On the third postoperative day, both the right control kidney and the left congested kidney were harvested and analyzed. Kidney weight and water content was increased, and renal injury and fibrosis were observed in the left congested kidney. Kidney weight gain and hydration were improved with tofogliflozin treatment. Additionally, this treatment effectively reduced renal injury and fibrosis, particularly in the renal cortex. SGLT2 expression was observed in the congested kidney, but suppressed in the damaged tubular cells. Molecules associated with inflammation were increased in the congested kidney and reversed by tofogliflozin treatment. Mitochondrial dysfunction provoked by renal congestion was also improved by tofogliflozin treatment. Tofogliflozin protects against renal damage induced by renal congestion. SGLT2 inhibitors could be a candidate strategy for renal impairment associated with heart failure.

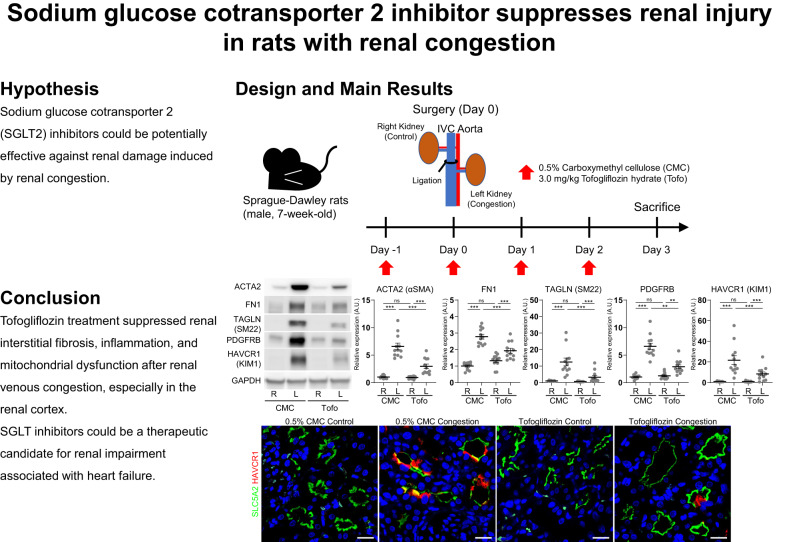

## Introduction

The kidneys and heart are closely related and have a specific characteristic known as cardiorenal syndrome, in which a dysfunction of one organ leads to a dysfunction of the other [1]. Patients with congestive heart failure have higher central and renal venous pressures than healthy subjects [2]. Renal venous congestion caused by elevated central and renal venous pressures is linked closely to cardiorenal syndrome and is responsible for the progression of heart failure [3–5]. Continuous elevation of central venous pressure is a strong indicator to predict worsening renal function in patients with heart failure [6]. Therefore, the management of renal congestion could be a therapeutic candidate in renal dysfunction and heart failure.

Analysis of venous congestion is difficult to evaluate in humans because of the concomitant reduction in renal blood flow due to low output or underlying chronic kidney disease. Therefore, animal models can be used to gain pathophysiological insight into renal congestion [7]. Acute increases in renal venous pressure caused by renal venous constriction result in elevated renal interstitial hydrostatic pressure and reduced renal blood flow, and subsequent sodium retention and diuresis [8–10]. Renal decongestion can ameliorate renal damage in high salt-loaded Dahl salt-sensitive rats [11, 12], which is a well-validated model of cardiorenal failure [13]. However, the mechanisms of renal congestion were not fully understood.

To investigate the pathophysiological role of renal congestion, we have created a novel experimental model of renal congestion in rats [14]. In this model, renal venous pressure in the left kidney was elevated by ligation of the inferior vena cava (IVC) between the renal veins, resulting in renal congestion in the left kidney only. Microarray analysis in this model revealed upregulation of extracellular factors, kidney injury markers, and genes in the focal adhesion pathway, and downregulation of genes in the electron transport chain pathway of mitochondria in the congested kidney [14]. Blockage of pericyte-myofibroblast transition (PMT) by inhibition of the platelet derived growth factor receptor (PDGFR) signaling pathway was also effective in our renal congestion, particularly in tubulointerstitial injury of the outer medulla [15]. Pericytes and the PDGFR pathway are normally involved in capillary homeostasis [16, 17] and activation of the PDGFR pathway induces PMT and renal fibrosis during progressive renal injury [18]. However other new strategies are needed for the protection of the cortex and mitochondria.

Sodium glucose cotransporter 2 (SGLT2, encoded by Solute Carrier Family 5 Member 2 (SLC5A2)) is expressed on the apical membrane of the S1 and S2 segments of the proximal tubule and contributes glucose and sodium reabsorption [19, 20]. Although SGLT2 inhibitors were initially developed as a new class of antidiabetic agents, recent accumulating evidence from both clinical and basic studies has shown the cardo-renal protective capacity of SGLT2 inhibitors through several mechanisms including diuretic and anti-inflammatory effects [21–26]. Furthermore, SGLT2 inhibitor blocks the transcriptional activity of osteopontin (OPN; also known as secreted phosphoprotein 1 (SPP1)) and mitochondrial reactive oxygen species (ROS) generation [27–29]. These previous reports suggest that SGLT2 inhibitors may have renoprotective benefits against several renal injuries. However, information on SGLT2 inhibitors for renal damage caused by renal congestion is limited.

We, therefore, hypothesized that SGLT2 inhibitors could be potentially effective against renal damage induced by renal congestion. To test this hypothesis, we examined the effectiveness of SGLT2 inhibitors on renal damage in our rat model of renal congestion.

## Methods

### Animals

All animal experiments conducted in this study were in accordance with the National Institutes of Health Guide for the Care and Use of Laboratory Animals, and were approved by the Animal Experiment Committee of Tohoku Medical and Pharmaceutical University (registration numbers: A18019-a, A19039-cn, A20005-cn, A21008-cn, and A22042-cn). Six-week-old male Sprague-Dawley (SD) rats were purchased from Japan SLC (Shizuoka, Japan). The rats were housed in environmentally controlled rooms with a 12-h light/dark cycle and free access to a standard chew (CE-2; CLEA Japan, Tokyo, Japan) and water. The animal experiment was performed after 1 week of acclimatization and designed to use all animals for the analysis, except those that died or had animal welfare issues.

### Renal congestion operation and pharmacological treatment

Thirty-eight male SD rats were randomly divided into groups with and without tofogliflozin treatment (Supplementary Fig. 1). Tofogliflozin hydrate, kindly provided by Kowa Company, Ltd, was dissolved in 0.5% carboxymethyl cellulose sodium salt (CMC; FUJIFILM Wako Pure Chemical). The treated rats received oral administration of tofogliflozin (3.0 mg/kg/day) from the day before until 2 days after IVC ligation surgery, while the non-treated rats received 0.5% CMC. The tofogliflozin hydrate dose was determined based on a previous report [30].

All rats underwent IVC ligation to induce renal congestion in the left kidney only, as described in our previous reports [14, 15]. Briefly, the IVC was ligated between the renal veins under mixed anesthesia with medetomidine (0.15 mg/kg body weight; Maruishi Pharmaceutical, Osaka, Japan), midazolam (2.0 mg/kg body weight; Astellas Pharma, Tokyo, Japan), and butorphanol (2.5 mg/kg body weight; Meiji Seika Pharma, Tokyo, Japan) on a 38 °C temperature-controlled surgical table. Three days after the IVC ligation, blood and urine were collected from the abdominal aorta and urinary bladder, respectively, under the above mixed anesthesia. The kidneys, heart, and liver were then isolated and weighed (*n* = 12 per group). The tissues for histological analysis were immediately fixed in 10% formalin (Mildform; Wako Pure Chemical Industries, Osaka, Japan) and embedded in paraffin. Both kidneys were dissected into the cortex and outer medulla, and stored in RNA-stabilizing solution (RNAlater solution; Invitrogen, Carlsbad, CA) for mRNA extraction or snap-frozen in liquid nitrogen for protein extraction. Biochemical examinations of the plasma and urine were performed in Nagahama Life Science Laboratory (Nagahama, Japan). The preanesthesia blood glucose levels were measured through tail vein bleeds with an experimental animal glucometer (SUGL-001; ForaCare, Tokyo, Japan).

### Measurement of renal water content

The whole kidneys (*n* = 7 per group) were weighed immediately after tissue isolation under the above mixed anesthesia as renal wet weight. Both kidneys were dried on sterile papers in an incubator (Mini Incubator, IC-150MA; AS ONE, Osaka, Japan) at 80 °C for at least 48 h until constant weight was observed. Then the weights were recorded as renal dry weight. The renal water content percentage was calculated using the following formula: Renal water content (%) = [(renal wet weight − renal dry weight)/renal wet weight] × 100.

### Quantitative reverse transcription-polymerase chain reaction

The expression levels of the mRNA in the kidney portion were quantified by reverse transcription real-time quantitative polymerase chain reaction (RT-qPCR) according to our previous reports [12, 15, 28]. In brief, total RNA was isolated using ISOGEN (NIPPON GENE, Tokyo, Japan) and cDNA was prepared using random hexamer (Invitrogen) and PrimeScript reverse transcriptase (TaKaRa Bio, Shiga, Japan). The cDNA of interest was amplified in duplicate on a commercial PCR instrument (CFX Connect Real-Time PCR Detection System; Bio-Rad, Hercules, CA) using gene-specific primers (Supplementary Table 1) and THUNDERBIRD Next SYBR qPCR Mix (TOYOBO, Osaka, Japan). The relative mRNA expression was standardized to the expression of *ribosomal protein lateral stalk subunit P2* (*Rplp2*), *peptidylprolyl isomerase A* (*Ppia*), and *phosphoglycerate kinase 1* (*Pgk1*).

### Western blotting

Western blotting was performed as previously described [12, 15, 28]. Briefly, rat kidney tissue was homogenized in cell lysis buffer (9803; Cell Signaling Technology, Danvers, MA) containing protease inhibitors. Protein concentration was determined by the Bradford method. Twenty micrograms of total protein in Laemmli sample buffer (Bio-Rad) and 2.5% 2-mercaptoethanol were separated on 4–20% polyacrylamide gel (Mini-PROTEAN TGX Precast Gel, Bio-Rad) and transferred to a polyvinylidene difluoride (PVDF) membrane (Bio-Rad). The membrane was incubated with primary antibodies (Supplementary Table 2) overnight at 4 °C after pre-treatment with blocking reagent (PVDF Blocking Reagent for Can Get Signal, TOYOBO) for 30 min at room temperature. The antibodies were visualized using horseradish peroxidase-conjugated secondary antibodies (1:5000; Cell Signaling) in an enhanced chemiluminescence system (Clarity Western ECL Substrate, Bio-Rad). Images were acquired with a chemiluminescence detection system (WSE-6300H LuminoGraph III; ATTO, Tokyo, Japan). Relative protein expression was normalized to glyceraldehyde-3-phosphate dehydrogenase (GAPDH) expression.

### Histological analysis

Formalin-fixed, paraffin-embedded tissues were sectioned at 4.0 µm thickness for immunostaining and routine histological analysis as described in our previous reports [12, 15, 28]. Hematoxylin-eosin, Elastica-Masson, and Elastica-Masson-Trichrome (EMT) staining were performed in the Technical Service Division of Tohoku Medical and Pharmaceutical University. Mitochondria structures were observed on EMT slides (*n* = 4 per group) using a confocal microscope (TCS-SP8; Leica Microsystems, Wetzlar, Germany) according to previously reported [28, 31]. The length of the mitochondria was measured using ImageJ.

For immunostaining, after deparaffinization and hydration, the antigen retrieval was performed by autoclaving for 5 min at 121 °C in 10 mmol/L citrate buffer (pH 6.0) or 1.0 mmol/L ethylenediaminetetraacetic acid buffer (pH 9.0). The slides were then incubated with primary antibodies (Supplementary Table 2) overnight at 4 °C. The following day, the slides were reacted with Histofine Simple Stain MAX PO (Nichirei Biosciences, Tokyo, Japan) or fluorophore (Alexa 488 or Alexa 555)-conjugated secondary antibodies (1:1000; Molecular Probes, Carlsbad, CA) for 30 min at room temperature. Immunostaining was developed by 3,3′-diaminobenzidine (Vector Laboratories, Newark, CA), counterstained with hematoxylin, and acquired by NanoZoomer-SQ (Hamamatsu Photonics, Hamamatsu, Japan). Immunofluorescent slides were counterstained with Hoechst 33342 (Molecular Probes) and captured by TCS-SP8. Three fields were randomly chosen in each rat and the percentage of Elastica-Masson and immunostaining positive area was quantified using ImageJ with a thresholding method. The individual rat value was obtained by calculating the average of the three fields.

### Low-vacuum scanning electron microscopy

The ultrastructure of the vasa recta was examined by low-vacuum scanning electron microscopy (LV-SEM, Miniscope TM4000; Hitachi High-Technologies, Tokyo, Japan) as previously reported [12, 14, 15]. Briefly, deparaffinized rat kidney slides (4.0 µm thick) were stained with Pt-blue solution (TI-blue small kit; Nisshin EM, Tokyo, Japan). Images were taken under the following conditions: acceleration voltage 15 kV and chamber pressure 30 Pa.

### Statistical analysis

Data were collected, interpreted, and statistically analyzed by separate investigators using blinded procedures. JMP Pro software (version 17.1.0; SAS Institute, Carry, NC) was used for statistical analysis. Continuous values are expressed as mean ± standard error of the mean (SEM). The Mann–Whitney *U* test was used for comparison between the two groups. The Kruskal–Wallis test was used to analyze 3 or more groups, followed by *post hoc* analysis using the Steel-Dwass test for multiple comparisons. A *P* value less than 0.05 was considered statistically significant.

## Results

### Biometric, biochemical, and morphological changes

No rats died or had any animal welfare problems during the experimental period. The weight and water content percentage in the left congested kidney were significantly higher than those in the right contralateral non-congested kidney (Table 1). Tofogliflozin treatment significantly reversed the increased weight and water content percentage in the congested kidney. Among renal function parameters, blood urea and serum creatinine were significantly improved by tofogliflozin treatment. Serum potassium levels were significantly decreased by tofogliflozin treatment. There was no statistical difference in other parameters between rats with and without tofogliflozin treatment. Serum glucose concentration collected from the abdominal aorta under the mixed anesthesia was approximately 250–70 mg/dL. It is likely that anesthesia, which is recommended for animal experiments in our animal facility, affected the level of serum glucose [32]. The preanesthetic blood glucose concentration from tail vein bleedings was 146.9 ± 4.4 mg/dL in tofogliflozin-treated rats and 140.7 ± 4.0 mg/dL in non-treated rats with no statistically significant difference between the two groups (*P* = 0.25). These values are consistent with the normal serum glucose levels in SD rats (approximately 100–50 mg/dL) [33]. Urinary glucose concentration was significantly higher in tofogliflozin-treated rats (124.2 ± 26.6 mg/dL) than in non-treated rats (11.4 ± 4.1 mg/dL). IVC ligation surgery or tofogliflozin treatment did not alter the morphology of the heart and liver (Table 1, Supplementary Fig. 2).

**Table 1:** Biometric and biochemical analysis

	0.5% CMC	Tofogliflozin
*n*	12	12
BW (mg)	233.1 ± 11.4	237.5 ± 13.0
Kidney (mg/g BW)^a^		
Right non-congested	4.48 ± 0.28	4.35 ± 0.31
Left congested	6.04 ± 0.57^†^	4.89 ± 0.46^†, #^
Renal water content (%)^a,b^		
Right non-congested	78.5 ± 0.5	78.2 ± 0.5
Left congested	85.8 ± 0.6^†^	82.4 ± 0.6^†, #^
Heart (mg/g BW)	3.28 ± 0.22	3.26 ± 0.16
Liver (mg/g BW)	40.6 ± 2.3	39.9 ± 2.6
Serum		
TP (g/dL)	5.15 ± 0.61	5.36 ± 0.40
Alb (g/dL)	3.20 ± 0.49	3.35 ± 0.24
BUN (mg/dL)	24.0 ± 0.46	19.9 ± 0.36*
Cr (mg/dL)	0.30 ± 0.03	0.26 ± 0.04*
UA (mg/dL)	1.22 ± 0.34	1.14 ± 0.33
Na (mEq/L)	136.8 ± 1.4	139.2 ± 0.7
K (mEq/L)	5.90 ± 0.26	4.88 ± 0.20*
Cl (mEq/L)	100.2 ± 0.8	99.5 ± 0.5
Ca (mEq/L)	10.4 ± 0.3	10.7 ± 0.2
IP (mg/dL)	8.1 ± 0.2	9.5 ± 0.2
T-cho (mg/dL)	68.3 ± 2.0	69.3 ± 2.0
Glu (mg/dL)	270.1 ± 11.5	291.3 ± 15.4

Values are mean ± SEM

*BW* body weight, *TP* total protein, *Alb* albumin, *BUN* blood urea nitrogen, *Cr* creatinine, *UA* uric acid, *Na* sodium, *K* potassium, *Cl* chloride, *Ca* calcium, *IP* inorganic phosphate, *T-Cho* total cholesterol

*P < 0.05 versus rats without tofogliflozin treatment (CMC) by Mann–Whitney *U* test

^†^*P* < 0.05 versus the right non-congested kidney in each group

^#^*P* < 0.05 versus the left congested kidney without tofogliflozin treatment by Steel-Dwass test

^a^Kruskal–Wallis test followed by Steel-Dwass post hoc test was used

^b^*n* = 7 per group

### Effect of tofogliflozin on renal injury and fibrosis

**Fig. 1:**
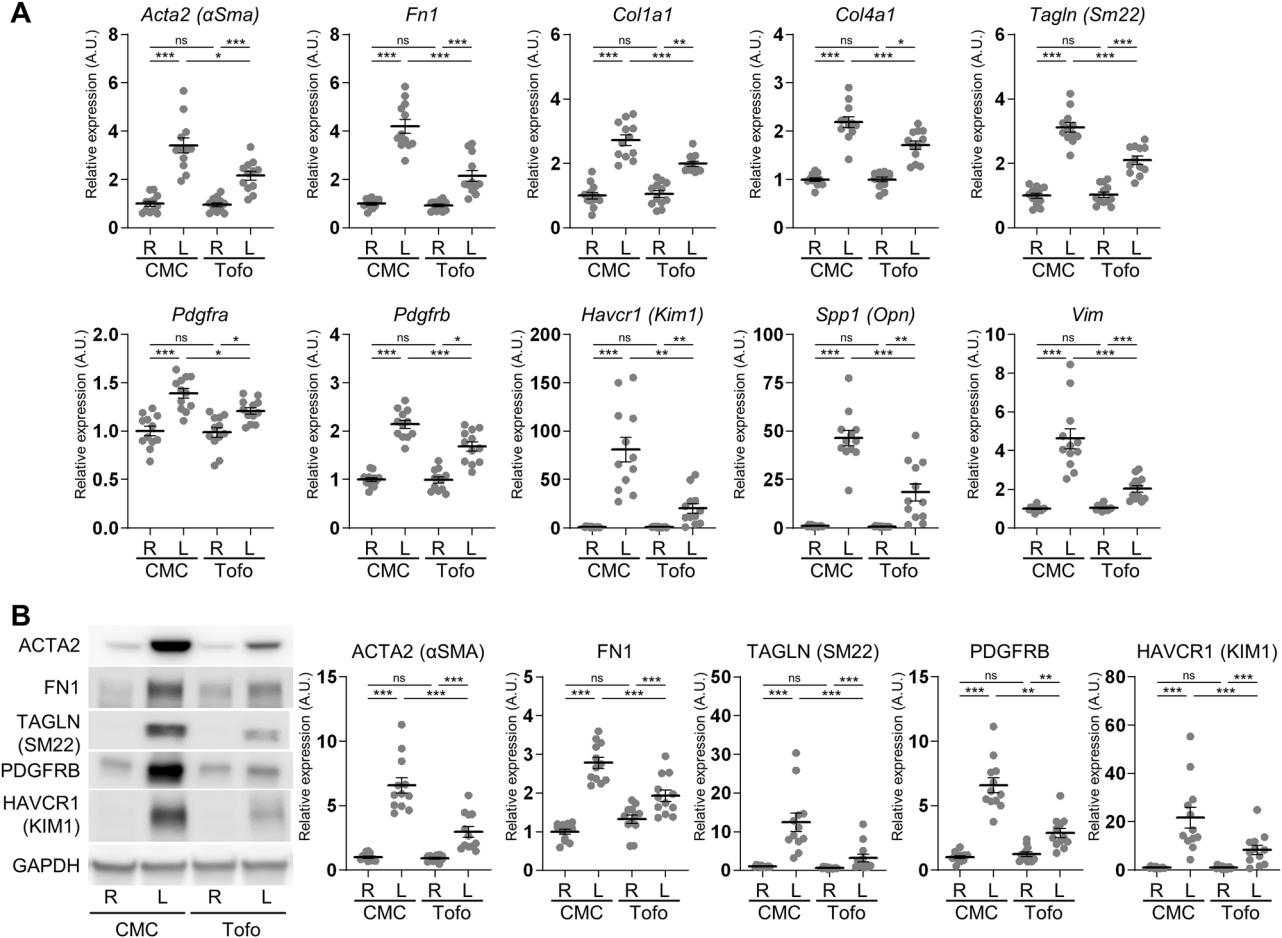
Molecular analysis of renal injury and fibrosis in the cortex of rats with IVC ligation. **A** The mRNA level of tubular injury and fibrosis-related genes was measured by reverse transcription real-time quantitative polymerase chain reaction in the cortex. The mRNA expression was normalized to *Rplp2*, *Ppia*, and *Pgk1*. **B** Representative immunoblotting and quantitative data of tubular injury and fibrosis-related proteins in the cortex. The protein expression was normalized to GAPDH. Data are presented as individual values and mean ± SEM; the relative value of the right contralateral non-congested kidney without tofogliflozin treatment was arbitrarily set to “1”; *n* = 12 per group. *P < 0.05, **P < 0.01, ***P < 0.001, ns not significant (*P* > 0.05) by Steel-Dwass test. R, right kidney; L, left kidney; CMC, rats without tofogliflozin treatment; Tofo, rats with tofogliflozin treatment; A.U., arbitrary unit

**Fig. 2:**
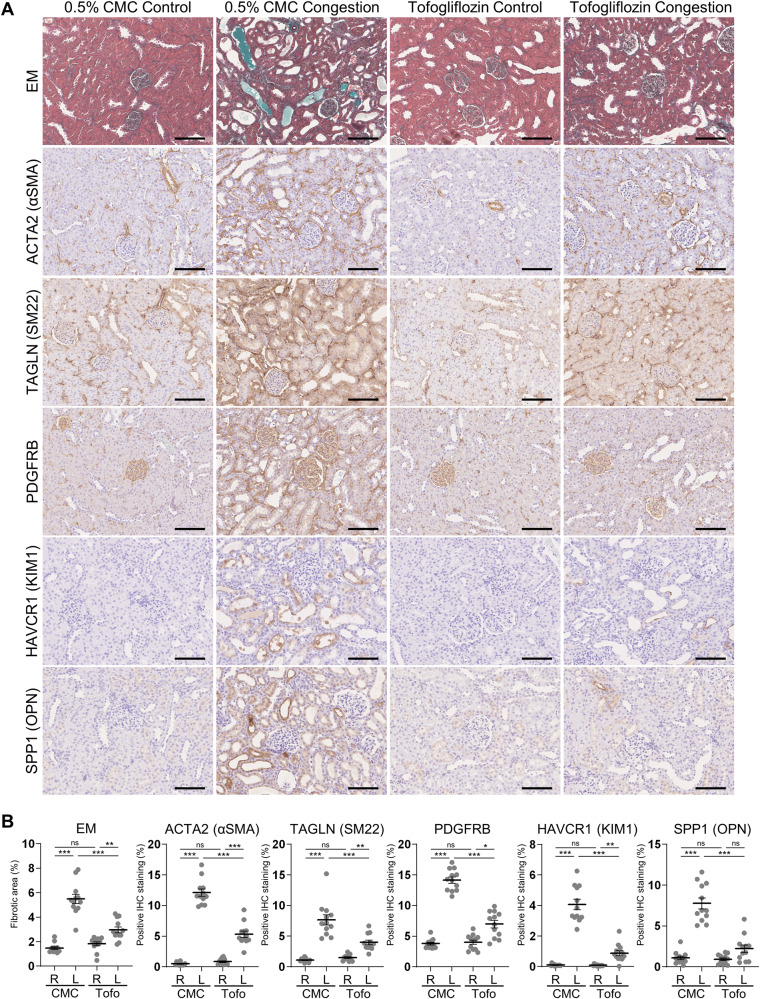
Histological analysis of renal injury and fibrosis in the cortex of rats with IVC ligation. **A** Representative light micrographs of Elastica-Masson (EM) staining and immunohistochemical staining for markers of fibrosis and tubular damage in the cortex. Scale bar = 100 µm. **B** Quantification of positively stained area of EM staining and immunohistochemical staining using ImageJ. Data are presented as individual values and mean ± SEM; *n* = 12 per group. *P < 0.05, **P < 0.01, ***P < 0.001, ns not significant (*P* > 0.05) by Steel-Dwass test. R, right kidney; L, left kidney; CMC, rats without tofogliflozin treatment; Tofo, rats with tofogliflozin treatment

In the cortex of rats without tofogliflozin treatment, the mRNA expression levels of the fibrosis markers *Actin alpha2* (*Acta2*; also known as *Alpha-smooth muscle actin* (*α-Sma*)), *Fibronectin* (*Fn1*), and collagens (*Col1a1* and *Col4a1*); the mesenchymal cell type markers *Transgelin* (*Tagln*; also known as *Smooth muscle protein 22-alpha* (*Sm22*)), *Pdgfra*, *Pdgfrb*, and *Vimentin* (*Vim*); and the tubular injury markers *Kidney injury molecule 1* (*Kim1*; also known as *hepatitis A virus cellular receptor 1* (*Havcr1*)) and *Spp1* were significantly upregulated in the left congested kidney, compared to the right contralateral non-congested kidney (Fig. 1A). Tofogliflozin treatment significantly attenuated the expression of these markers in the left congested kidney. Similarly, the protein expression of these markers ACTA2, FN1, TAGLN, PDGFRB, and KIM1 was significantly elevated in the cortex of the congested kidney, and was suppressed by tofogliflozin treatment (Fig. 1B). Histological analysis revealed hyperplasia of the extracellular matrix, renal fibrosis, and tubular injury in the cortex of the congested kidney, which were reduced by tofogliflozin treatment (Fig. 2A). Elastica-Masson staining showed increased interstitial fibrosis and tubular casts in the congested kidney (Fig. 2B). The positive area of ACTA2, TAGLN, PDGFRB, KIM1, and OPN was significantly increased in the congested kidney (Fig. 2B). Tofogliflozin treatment improved these areas, similar to the results for mRNA and protein expression. In contrast to the cortex, the effect of tofogliflozin was limited in the outer medulla (Supplementary Figs. 3 and 4). Vasa recta expansion and pericyte detachment, which are triggers of renal damage in our renal congestion model [14, 15] and observed in heart failure patients [12], were not altered in rats with and without tofogliflozin treatment in the ultrastructural image of the outer medulla captured by LV-SEM (Supplementary Fig. 5).

### Alteration of SGLT2 expression

**Fig. 3:**
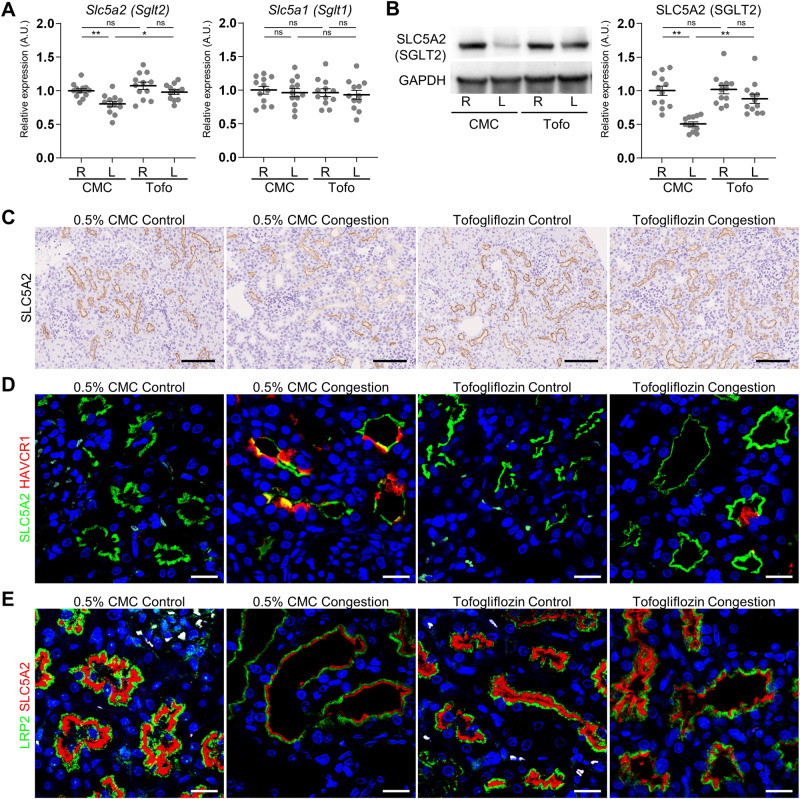
SGLT1 and SGLT2 expression in the cortex of rats with IVC ligation. **A** The mRNA level of *Slc5a1* (*Sglt1*) and *Slc5a2* (*Sglt2*) was measured by reverse transcription real-time quantitative polymerase chain reaction in the cortex. The mRNA expression was normalized to *Rplp2*, *Ppia*, and *Pgk1*. **B** Representative immunoblotting and quantitative data of SLC5A2 (SGLT2) in the cortex. The protein expression was normalized to GAPDH. Data are presented as individual values and mean ± SEM; the relative value of the right contralateral non-congested kidney without tofogliflozin treatment was arbitrarily set to “1”; *n* = 12 per group. *P < 0.05, **P < 0.01, ***P < 0.001, ns not significant (*P* > 0.05) by Steel-Dwass test. R, right kidney; L, left kidney; CMC, rats without tofogliflozin treatment; Tofo, rats with tofogliflozin treatment, A.U., arbitrary unit. **C** Representative light micrographs of immunohistochemical staining for SGLT2 in the cortex. Scale bar = 100 µm. The corresponding figures are provided in Supplementary Fig. S6. Representative confocal micrographs of double immunofluorescence staining for **D** SLC5A2 (SGLT2; green) and tubular injury marker HAVCR1 (KIM1; red) and **E** SLC5A2 (SGLT2; red) and proximal tubules marker LRP2 (megalin; green). The nuclei were counterstained with Hoechst 33342 (blue). Scale bar = 20 µm

The SGLT2 gene and protein expression was significantly decreased in the cortex of the left congested kidney without tofogliflozin treatment, and significantly recovered by tofogliflozin treatment (Fig. 3A, B). In contrast, the SGLT1 gene expression was not altered by IVC ligation surgery or tofogliflozin treatment. The SGLT2 immunoreactivity was observed in the apical membrane of the proximal tubules in all experimental groups (Fig. 3C, Supplementary Fig. 6). This immunoreactivity was partially suppressed in the congested kidney. Furthermore, the SGLT2 immunofluorescence was weak or undetectable in KIM1-positive damaged proximal tubular cells (Fig. 3D). The immunofluorescence intensity of SGLT2 was become weaker in parallel to that of LRP2 (Fig. 3E).

### Tofogliflozin suppresses inflammation

**Fig. 4:**
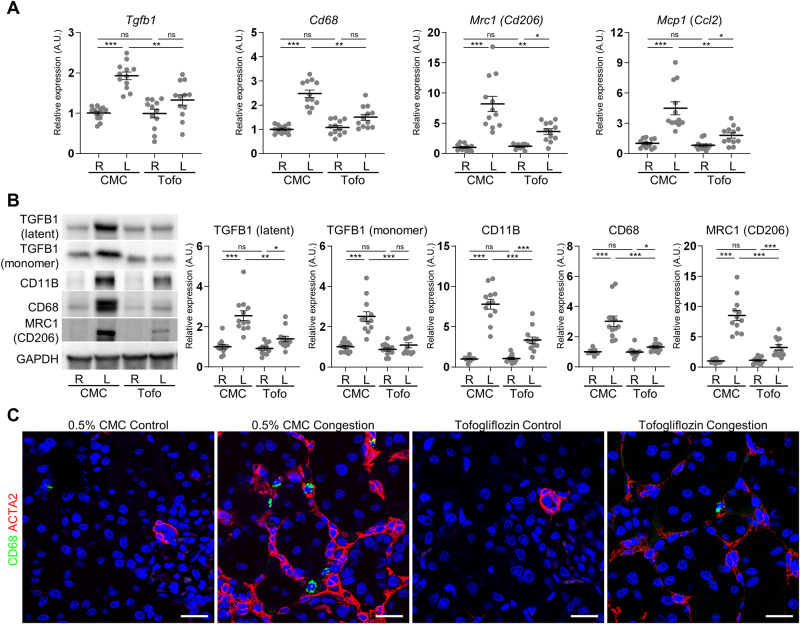
Effect of tofogliflozin on TGFB and macrophage markers. **A** The mRNA level of proinflammatory chemokine and cytokine, and macrophage-related genes was measured by reverse transcription real-time quantitative polymerase chain reaction in the cortex. The mRNA expression was normalized to *Rplp2*, *Ppia*, and *Pgk1*. **B** Representative immunoblotting and quantitative data of TGFB1 and macrophage markers in the cortex. The protein expression was normalized to GAPDH. Data are presented as individual values and mean ± SEM; the relative value of the right contralateral non-congested kidney without tofogliflozin treatment was arbitrarily set to “1”; *n* = 12 per group. *P < 0.05, **P < 0.01, ***P < 0.001, ns: not significant (*P* > 0.05) by Steel-Dwass test. R, right kidney; L, left kidney; CMC, rats without tofogliflozin treatment; Tofo, rats with tofogliflozin treatment; A.U., arbitrary unit. **C** Representative confocal micrographs of double immunofluorescence staining for macrophage marker CD68 (green) and fibrosis marker ACTA2 (red) in the cortex. The nuclei were counterstained with Hoechst 33342 (blue). Scale bar = 20 µm

Renal congestion significantly induced the mRNA expression of the profibrotic mediator *Transforming growth factor beta 1* (*Tgfb1*); macrophage markers *Cd68* and *Cd206* (also known as *Mannose receptor C-type 1* (*Mrc1*)); and proinflammatory chemokine and cytokine chemokine (C-C motif) ligand 2 (Ccl2; also known as Monocyte chemoattractant protein 1 (*Mcp1*)) in the cortex (Fig. 4A). The protein expression of TGFB1, CD11B, CD68, and CD206 was also elevated by renal congestion (Fig. 4B). Double-labeling immunofluorescence analysis showed that ACTA2-positive interstitial fibrosis was observed around CD68-positive macrophage, but positive area of ACTA2 and CD68 did not colocalize (Fig. 4C). Tofogliflozin treatment suppressed these renal congestion-induced mRNA and protein expressions.

### Tofogliflozin recovers mitochondria

**Fig. 5:**
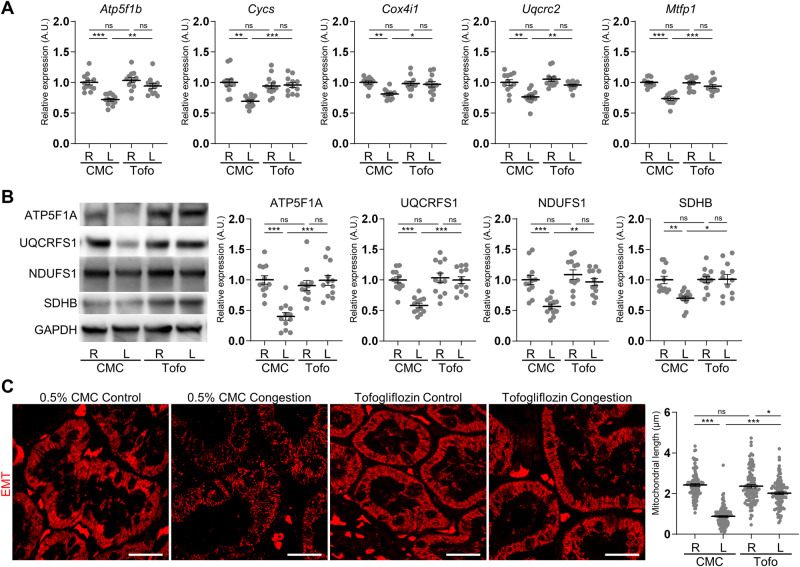
Effect of tofogliflozin on mitochondria markers. **A** The mRNA level of mitochondria-related genes was measured by reverse transcription real-time quantitative polymerase chain reaction in the cortex. The mRNA expression was normalized to *Rplp2*, *Ppia*, and *Pgk1*. **B** Representative immunoblotting and quantitative data of mitochondria markers in the cortex. The protein expression was normalized to GAPDH. Data are presented as individual values and mean ± SEM; the relative value of the right contralateral non-congested kidney without tofogliflozin treatment was arbitrarily set to “1”; *n* = 12 per group. *P < 0.05, **P < 0.01, ***P < 0.001, ns: not significant (*P* > 0.05) by Steel-Dwass test. R, right kidney, L, left kidney; CMC, rats without tofogliflozin treatment; Tofo, rats with tofogliflozin treatment; A.U. arbitrary unit. **C** Representative confocal micrographs and quantified length of mitochondria in proximal tubular cells. Images of Elastica-Masson Trichrome (EMT)-stained kidney sections excited at 552 nm were pseudo-colored in red. Data are presented as individual values and mean ± SEM; *n* = 109 (0.5% CMC Control), 114 (0.5% CMC Congestion), 101 (Tofogliflozin Control), and 104 (Tofogliflozin Congestion) from 4 fields per group. *P < 0.05, **P < 0.01, ***P < 0.001, ns: not significant (*P* > 0.05) by Steel-Dwass test. R, right kidney; L, left kidney; CMC, rats without tofogliflozin treatment; Tofo, rats with tofogliflozin treatment. Scale bar = 20 µm

Renal congestion significantly downregulated the mRNA expression of the mitochondria markers *ATP synthase F1 subunit beta* (*Atp5f1b*), *Cytochrome c somatic* (*Cycs*), *Cytochrome c oxidase subunit 4i1* (*Cox4i1*), *Ubiquinol cytochrome c reductase core protein 2* (*Uqcrc2*), and *Mitochondrial fission process 1* (*Mtfp1*), and tofogliflozin treatment recovered these expressions in the cortex (Fig. 5A). Similar to the mRNA expression, the protein levels of the mitochondria markers ATP5F1A, Ubiquinol Cytochrome C Reductase Rieske Iron–Sulfur Polypeptide (UQCRFS1), NADH Ubiquinone Oxidoreductase Core Subunit S1 (NDUFS1), and Succinate Dehydrogenase Complex Iron Sulfur Subunit B (SDHB) were decreased by renal congestion, and improved by tofogliflozin treatment (Fig. 5B). Furthermore, long tube-like mitochondrial structure was observed in the proximal tubules of the right contralateral control kidney (Fig. 5C). Renal congestion altered the structure form long tubular to globular shape. Mitochondria length was significantly shortened in the congested kidney. Tofogliflozin treatment restored this structure.

## Discussion

The present study showed that SGLT2 protein was still expressed in the apical membrane of non-injured proximal tubules after renal congestion and that an SGLT2 inhibitor tofogliflozin showed renoprotective effects against renal damage induced by renal congestion. Renal interstitial fibrosis, inflammation and mitochondrial dysfunction were reversed by tofogliflozin treatment. These results suggest that SGLT2 inhibitors are responsible for renal damage induced by renal congestion.

The expression of SGLT2 was decreased in the proximal tubules of the congested kidney in the present study. The expression pattern of SGLT2 differs in different types of kidney disorders. SGLT2 expression is upregulated in diabetic patients and animal models [34–37]. In contrast to diabetic nephropathy, its expression is decreased in models of the ischemia-reperfusion acute kidney injury [38], unilateral ureteral obstruction [39], and calcium-oxalate nephrolithiasis [28]. Furthermore, double-labeling immunofluorescence analysis revealed that SGLT2 expression was abolished or decreased in KIM1-positive damaged proximal tubules of the congested kidney, which is consistent with our previous report in calcium-oxalate nephrolithiasis rats [28]. Tubular injury causes loss of renal tubular transporters, including SGLT2 [39]. These results may suggest that the renoprotective potential of SGLT2 inhibitors may be limited in the injured proximal tubules. However, SGLT2 inhibitors show renoprotective ability in patients with chronic kidney disease with and without diabetes [21, 40]. Renoprotective effects are also observed in mice that started SGLT2 inhibitor treatment after ischemia-reperfusion [38]. Therefore, further investigation may be needed to clarify what level of renal function is required to obtain the renoprotective effect of SGLT2 inhibitors.

Renal congestion forms a vicious cycle of several factors including hormonal activation, inflammation, oxidative stress, sodium reabsorption, and volume overload [41, 42]. Several approaches are necessary to dissect the pathophysiological mechanisms relating to renal congestion. The IVC pressure on day 3 was approximately 10 mmHg in our renal congestion model [14, 15]. This pressure is comparable to the other renal congestion model in Dahl salt-sensitive rats fed high salt, in which the IVC pressure reached 12.2 mmHg [11]. In contrast to Dahl salt-sensitive rats, our rats with renal congestion have a contralateral control kidney that exhibits unchanged morphology and function as compared to sham-operated kidney [14]. Therefore, our model could have the potential to clarify the direct impact of renal congestion, without the influence of neuro/hormonal transmitters.

Increased kidney weight and water content, and progression of renal injury and fibrosis were observed in the congested kidney in the present study. They were reversed by tofogliflozin treatment. This may reflect renal decongestion and subsequent renoprotection by the SGLT2 inhibitor. SGLT2 inhibitors show renoprotective properties in Dahl salt-sensitive rats with high salt diet [43, 44]. SGLT2 inhibitors relieved renal congestion by removing excess fluid from both intravascular and renal medullary interstitial space, and suppressed renal intra-tubular cast formation in Dahl salt-sensitive rats fed high salt [43]. In contrast to Dahl salt-sensitive rats, the renoprotective effects of tofogliflozin treatment on medullary regions were limited by molecular and histological analysis in this study. Furthermore, tofogliflozin treatment recovered the upregulation of inflammatory molecules and mitochondrial dysfunction, which were reported in diabetic conditions but not in renal congestion including Dahl salt-sensitive rats fed high salt [45, 46]. Thus, the direct effect of SGLT2 inhibitors on the proximal tubules may be preferential in our renal congestion model.

The molecules associated with renal injury and fibrosis were upregulated in the left congested kidney and reversed by tofogliflozin treatment. Among them, OPN is a marker reflecting tubular injury and dramatically upregulated in the cortex of the congested kidney [14]. OPN is a secreted glycoprotein and expresses in the ascending lib of loop of Henle and distal tubules in normal kidneys in both humans and animals [47]. After several damages, its expression is highly upregulated in the proximal tubules in humans and rodents [48, 49]. OPN is also essential for TGFB1-mediated myofibroblast differentiation and activity [50]. In addition, SGLT2 inhibitors suppress the transcriptional activity of OPN by inhibiting glucose uptake in damaged proximal tubules [27]. Therefore, OPN and its suppression by SGLT2 inhibitors may be crucial targets to regulate fibrosis and the TGFB1 driven inflammatory cascade in the injured proximal tubular cells.

Myofibroblasts have a pivotal role in renal fibrosis, but the origin of these cells is still debated. Myofibroblasts are a heterogeneous population with a variety of origins, including tubular epithelial cells by epithelial-mesenchymal transition [51, 52], endothelial cells by endothelial-mesenchymal transition (EndoMT) [53] and pericyte cells by PMT [14, 15, 18, 54]. Indeed, suppression of PMT by inhibition of the PDGFR pathway reduced renal congestion-induced renal interstitial fibrosis in our previous study [15]. Furthermore, macrophage-myofibroblast transition is another source of myofibroblasts [55]. Approximately one-third of the myofibroblasts are derived from bone marrow cells in the unilateral ureteral obstruction model [56] and the renal allograft [57]. However, the macrophage marker (CD68) and myofibroblast marker (ACTA2) did not colocalize in the congested kidney of the present study. The contribution of macrophage-myofibroblast transition seems to be limited in renal fibrosis after renal congestion.

Mitochondrial dysfunction, previously observed in this model of renal congestion by transmission electron microscopy [14], was reversed by tofogliflozin treatment. The kidneys are enriched in mitochondria, second only to the heart [58, 59]. SGLT2 inhibitors recovered mitochondrial dysfunction in diabetic patients and mice [37, 45]. Glucose-induced toxicity leads to oxidative stress and mitochondrial dysfunction, which impar tubular function in diabetic kidney [60]. SGLT2 inhibitors improve mitochondrial number and mitophagy in cardiomyocytes [61]. Thus, SGLT2 inhibitors may also exert renoprotective effects by reversing mitochondrial dysfunction in renal congestion.

There are several limitations. First, the duration of treatment is important in assessing the therapeutic potential of SGLT2 inhibitors. It is difficult to start therapeutic intervention before the onset of renal congestion in patients. We started tofogliflozin treatment before IVC ligation surgery and evaluated it 3 days after the induction of renal congestion, which was a semi-acute phase. Because the formation of collateral circulation and attenuation of renal venous congestion occurred within 7 days after IVC ligation [14, 62], our model may not be suitable for chronic phase experiments. Further experiments using other models of renal congestion including Dhal salt-sensitive rats with high salt loading and deoxycorticosterone acetate (DOCA)-salt rats may be needed to evaluate the effects of SGLT2 inhibitors on long-term renal congestion and after renal congestion has occurred. Second, the mixed anesthesia, which is recommended for animal experiments in our animal facility, transiently increases serum glucose, hematocrit, and hemoglobin levels and decreases total protein levels and white blood cell counts, with most of these parameters returning to normal within 24 h [32]. In the present study, rats treated with and without tofogliflozin had blood glucose levels of approximately 140 mg/dL before being anesthetized for sacrifice, which is consistent with previous reports using tofogliflozin in normal rats [30, 33]. However, because these factors are closely related to the effects of SGLT2 inhibitors, anesthesia may influence the results of this study.

## Conclusion

Tofogliflozin treatment suppressed renal interstitial fibrosis, inflammation, and mitochondrial dysfunction after renal venous congestion, especially in the renal cortex. Therefore, SGLT2 inhibitors could be a therapeutic candidate for renal impairment associated with heart failure.

### Supplementary information

Supplementary information
