# Supplementary material for: Sodium glucose cotransporter 2 inhibitor suppresses renal injury in rats with renal congestion
Source: Hypertens Res. 2023 Sep 25;47(1):33–45. doi: 10.1038/s41440-023-01437-1 (PMC10766540; doi:10.1038/s41440-023-01437-1)
Supplement: Supplementary file 1 — Supplementary information [file 41440_2023_1437_MOESM1_ESM.pdf]

Supplementary information

Sodium glucose cotransporter 2 inhibitor suppresses renal injury in rats with renal congestion

Akari Endo[1,2], Takuo Hirose[1,2,3,*], Shigemitsu Sato[3], Hiroki Ito[1,2], Chika Takahashi[3], Risa Ishikawa[1], Ayaka Kamada[1], Ikuko Oba-Yabana[1], Tomoyoshi Kimura[1], Kazuhiro Takahashi[2], Takefumi Mori[1,3,*]

1 Division of Nephrology and Endocrinology, Faculty of Medicine, Tohoku Medical and Pharmaceutical University, Sendai, Japan
2 Department of Endocrinology and Applied Medical Science, Tohoku University Graduate School of Medicine, Sendai, Japan
3 Division of Integrative Renal Replacement Therapy, Faculty of Medicine, Tohoku Medical and Pharmaceutical University, Sendai, Japan

Running head: renal congestion and SGLT2 inhibitor

*Corresponding author
Takuo Hirose, Ph.D.
Department of Endocrinology and Applied Medical Science
Tohoku University Graduate School of Medicine
2-1, Seiryo, Aoba, 980-8575 Sendai, Japan
Tel/Fax: +81-22-717-7482
E-mail: hirose-t@med.tohoku.ac.jp
ORCID: 0000-0002-8761-3779

Takefumi Mori, M.D., Ph.D.
Division of Nephrology and Endocrinology, Faculty of Medicine,
Tohoku Medical and Pharmaceutical University
1-15-1, Fukumuro, Miyagino, 983-8536, Sendai, Japan
Tel: +81–22–259–1221
Fax: +81–22–259–1232
E-mail: tmori@tohoku-mpu.ac.jp

Supplementary Figure S1

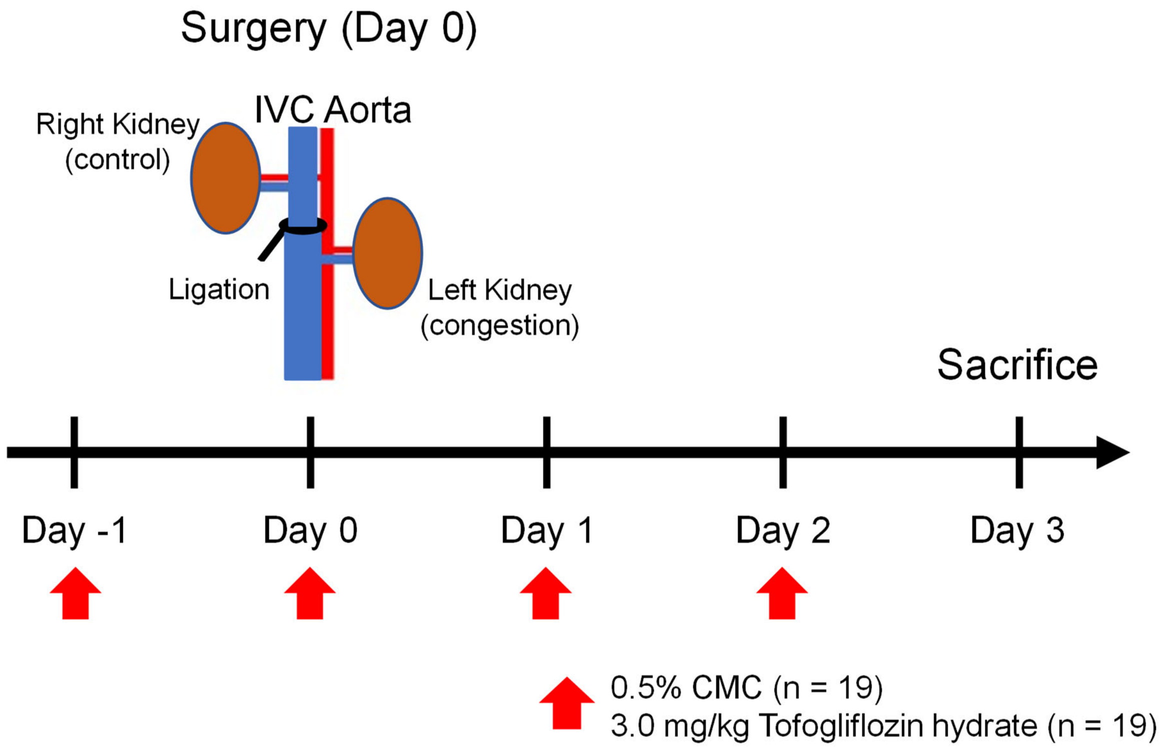

Supplementary Figure S1. Experimental design. Male Sprague-Dawley rats were divided into two groups: tofogliflozin-treated group (n = 19) and non-treated group (n = 19). The inferior vena cava (IVC) was ligated between renal veins on a 38°C temperature-controlled surgical table (Day 0). The treated rats received oral administration of tofogliflozin (3.0 mg/kg/day) from the day before until 2 days after IVC ligation surgery (red arrow), while the non-treated rats received 0.5% carboxymethyl cellulose sodium salt (0.5% CMC). Tissues (n = 12 in each group) were collected after blood and urine sampling, and rats were euthanized under deep anesthesia (Day 3). The remaining rats (n = 7 in each group) were used for renal water content measurement on day 3. No anatomical vein abnormalities were observed, and no rats died during the experimental period.

Supplementary Figure S2

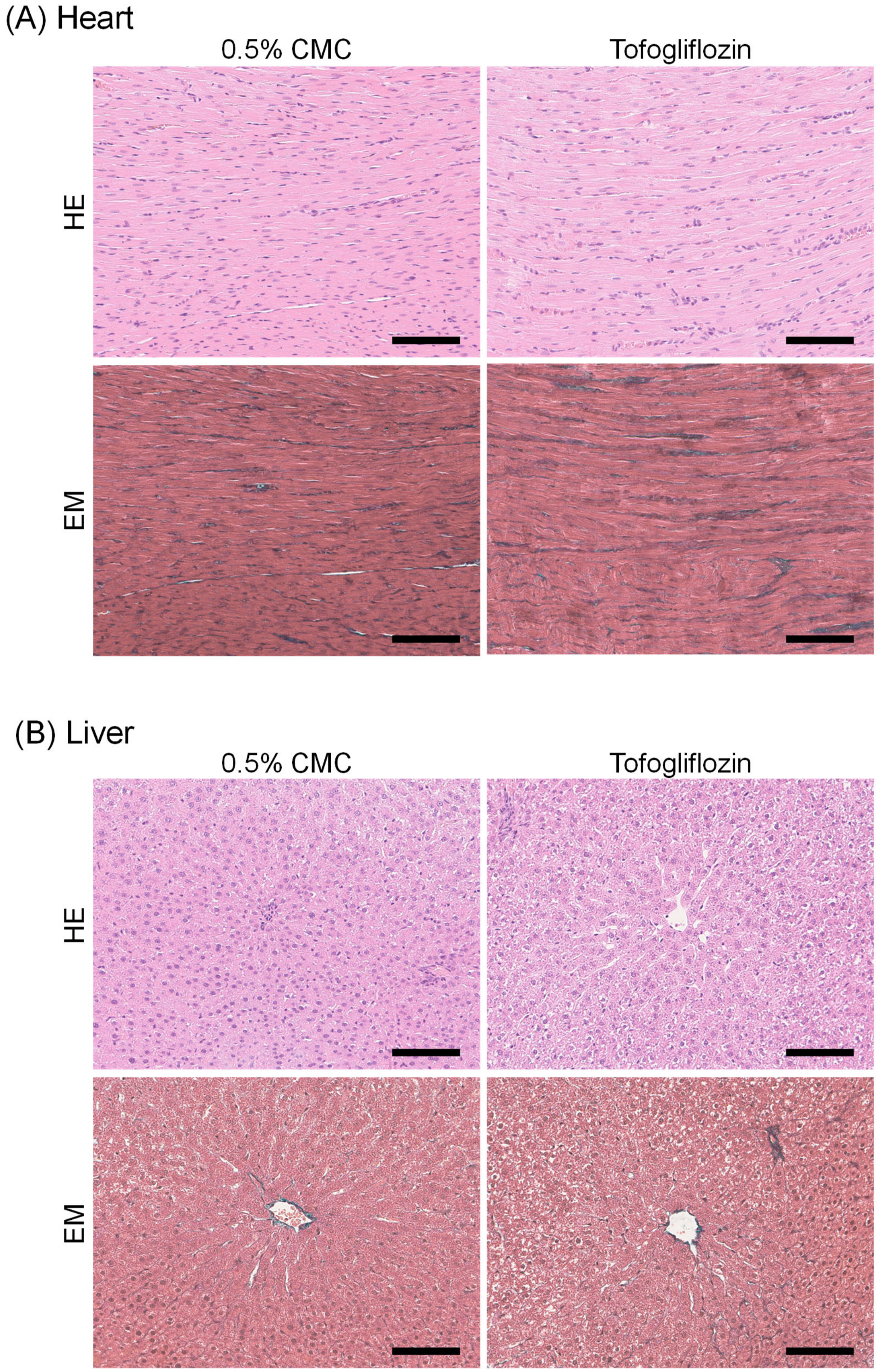

Supplementary Figure S2. Representative images of rat heart (**A**) and liver (**B**) stained with hematoxylin-eosin (HE) and Elastica-Masson (EM). The organs not affected by IVC ligation surgery or tofogliflozin treatment. Scale bar = 100 μm.

Supplementary Figure S3

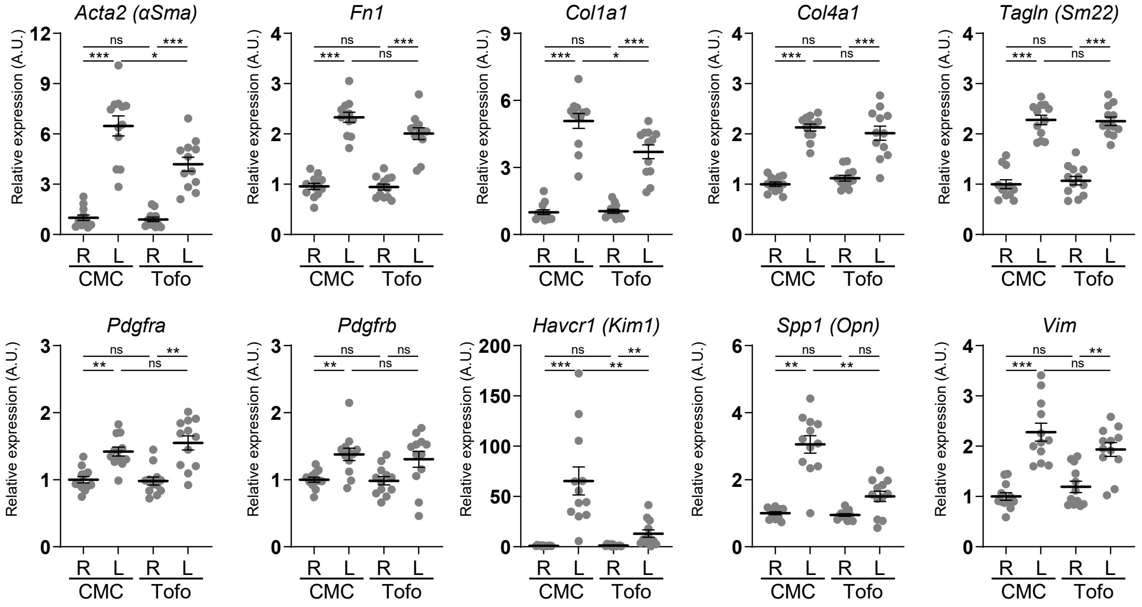

Supplementary Figure S3. Molecular analysis of renal injury and fibrosis in the outer medulla of rats with IVC ligation. The mRNA level of tubular injury and fibrosis-related genes was measured by reverse transcription real-time quantitative polymerase chain reaction in the cortex. The mRNA expression was normalized to *Rplp2*, *Ppia*, and *Pgk1*. Data are presented as individual values and mean ± SEM; the relative value of the right contralateral non-congested kidney without tofogliflozin treatment was arbitrarily set to "1"; n = 12 per group. *P<0.05, **P<0.01, ***P<0.001, ns: not significant (P>0.05) by the Steel-Dwass test. R, right kidney; L, left kidney; CMC, rats without tofogliflozin treatment; Tofo, rats with tofogliflozin treatment; A.U., arbitrary unit.

Supplementary Figure S4

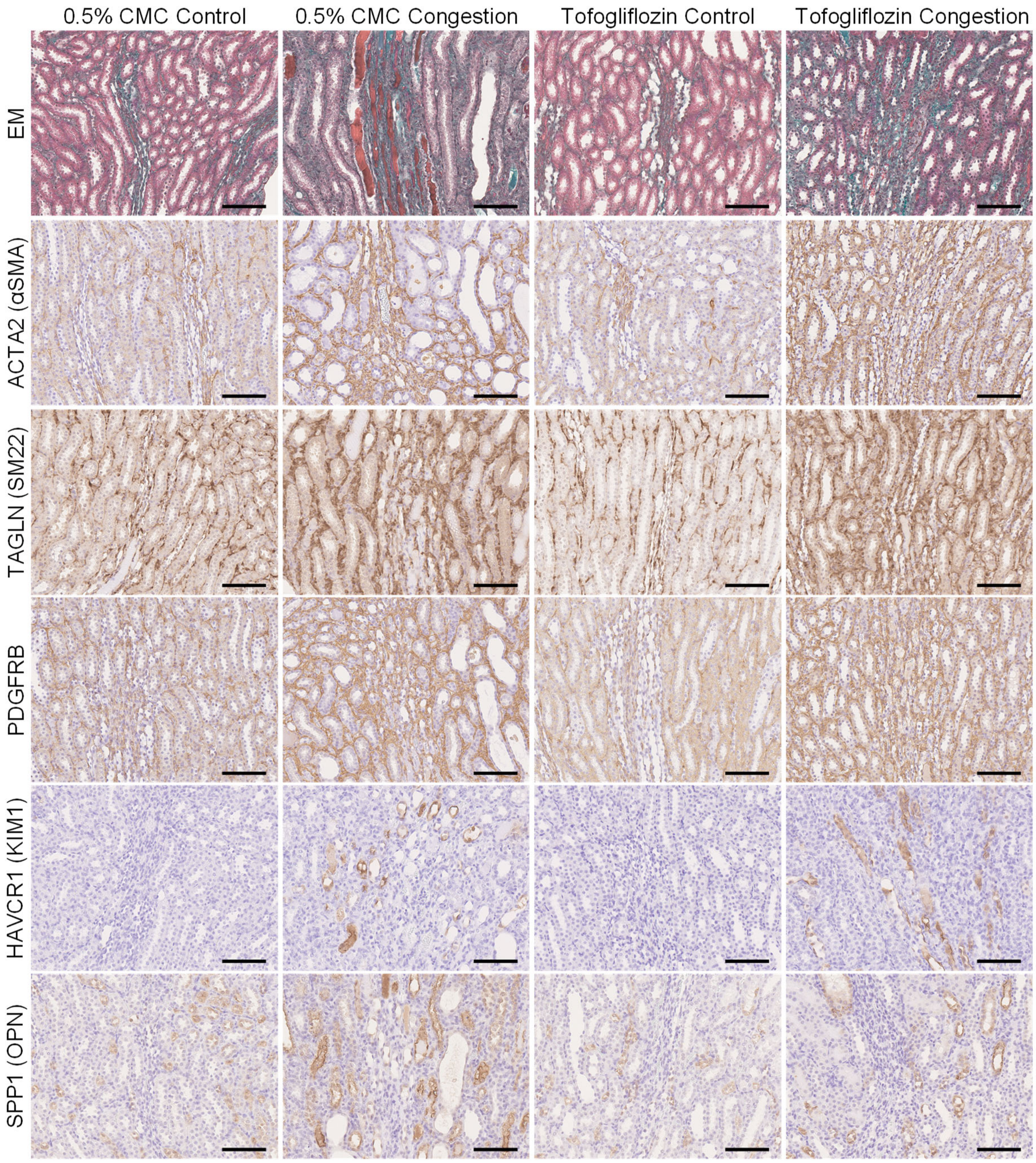

Supplementary Figure S4. Histological analysis of renal injury and fibrosis in the outer medulla of rats with IVC ligation. Representative light micrographs of Elastica-Masson staining (EM) and immunohistochemical staining for the markers of fibrosis and tubular damage in the outer medulla. Scale bar = 100 μm.

Supplementary Figure S5

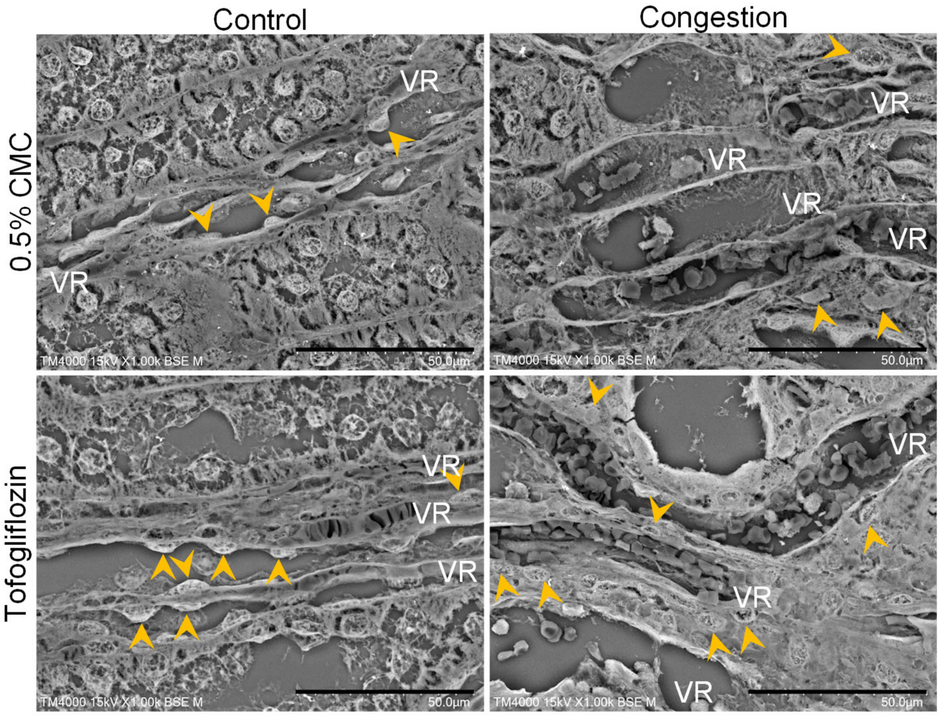

Supplementary Figure S5. Pericyte structure (arrowhead) in the descending vasa recta (VR) by low-vacuum scanning electron microscopy. Scale bar = 50 μm.

Supplementary Figure S6

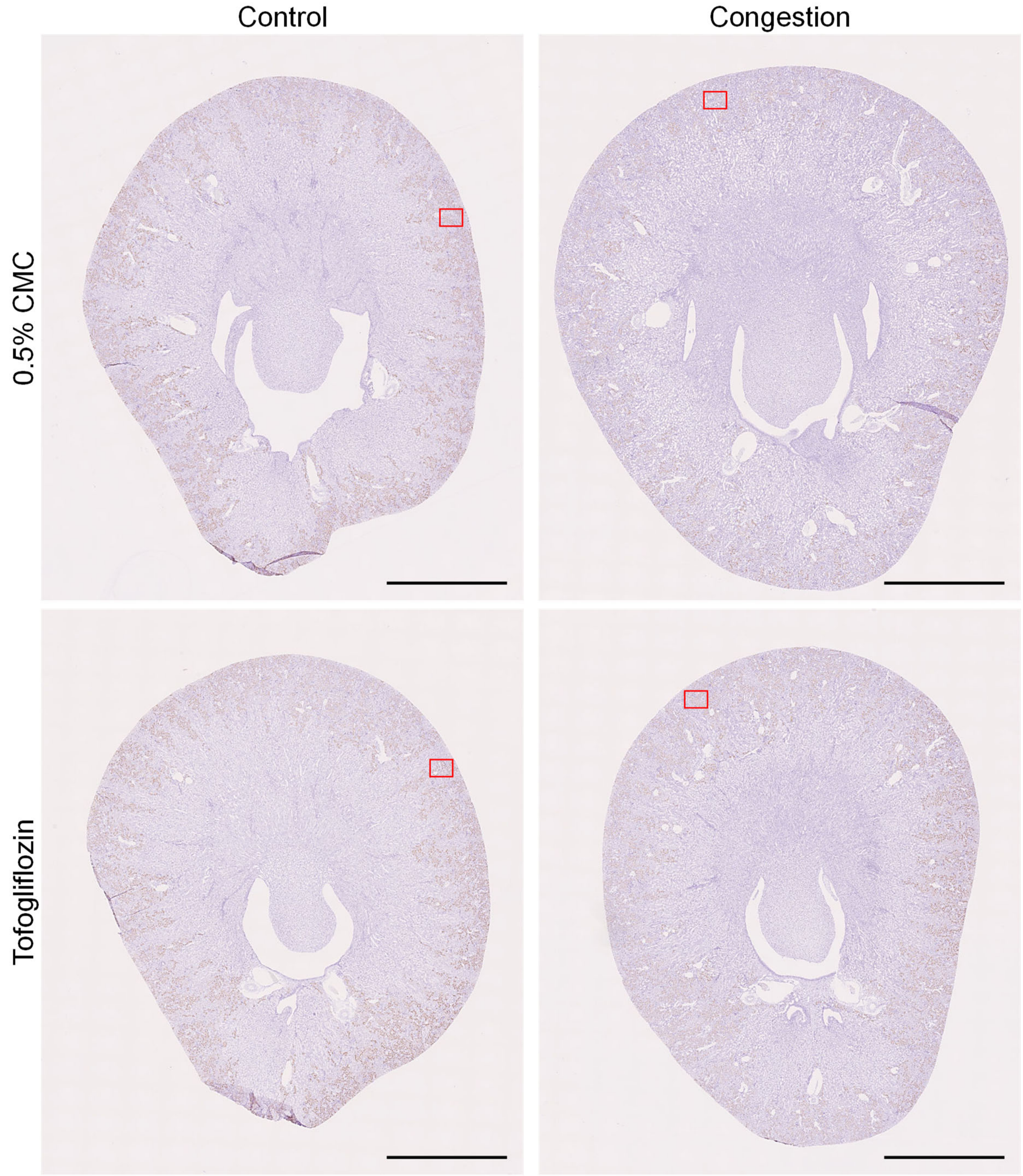

Supplementary Figure S6. Representative light micrographs of immunohistochemical staining for SGLT2. Red box indicates the area shown in Fig. 3C. Scale bar = 2.5 mm.

Supplementary Table S1: Primer information.

Symbol	primer set ID* or Forward primer / Reverse primer (5’-3’)**
Acta2 (*αSMA*)	RA060203
Atp5a1b	CCAAGGGTGGGAAAATCGGA / GGTCCTCTCACCAACACCAG
Ccl2 (*Mcp1*)	RA011410
Cox4i1	AATGTTGGCTACCAGGGCAC / GGGTAGTCACGCCGATCAAC
Cycs	GAAGAAGGGAGAAAGGGCAGA / ACTTAAATCGGGGCTGTCCA
Cd68	TGGATTCAAACAGGACCGAC / TGCGCTGAGAATGTCCACTG
Col1a1	RA065609
Col4a1	RA064189
Fn1	RA055827
Havcr1 (*Kim1*)	RA057664
Mrc1 (Cd206)	RA071208
Mtfp1	RA064596
Pdgfra	RA059727
Pdgfrb	RA048695
Pgk1	ATGGAGCCAAGTCGGTTGTG / GGTTGGCACAGGCATTCTCT
Ppia	CCACCGTGTTCTTCGACATC / CGTGTGAAGTCACCACCCTG
Rplp2	RA015377
Slc5a1 (*Sglt1*)	RA029236
Slc5a2 (*Sglt2*)	RA045566
Spp1 (*Opn*)	RA017345
Tagln (*Sm22*)	RA063024
Tgfb1	RA010073
Uqcrc2	ATGGTTTGGTGATTGCTTCTCTG / ACCAACTGCTTCAATTCCACG
Vim	RA050877

*RA XXs was purchased from Takara Bio (Shiga, Japan).

**Primers were designed by Primer3 (http://primer3.ut.ee) and synthesized by Integrated DNA Technologies (Coralville, IA).

Supplementary Table S2: Antibody information.

Antigen	Company	Catalog #	Clone	Host	WB	IHC	IF
ACTA2 (αSMA)	Cell Signaling	19245S	D4K9N	rabbit	1:1000	1:500**	1:200**
ATP5A1	Cell Signaling	18023		rabbit	1:1000		
CD11B	Abcam	ab133357	EPR1344	rabbit	1:1000		
CD68	Bio-Rad	MCA341R	ED1	mouse	1:1000		1:2000**
FN1	Merck	F3648		rabbit	1:1000		
GAPDH	Cell Signaling	2118S	14C10	rabbit	1:5000		
HAVCR1 (KIM1)	R&D system	AF3689		goat	1:1000	1:1250*	1:1250**
LRP2 (Megalin)	Santa Cruz	sc-515772	H-10	mouse			1:2000**
MRC1 (CD206)	Cell Signaling	24595	E6T5J	rabbit	1:1000		
NDUFS1	Cell Signaling	70264	E4K3E	rabbit	1:1000		
PDGFRB	Abcam	ab32570	Y92	rabbit	1:1000	1:200**	
SDHB	Cell Signaling	92649	E3H9Z	rabbit	1:1000		
SLC5A2 (SGLT2)	Abcam	ab85626		rabbit	1:1000		
SLC5A2 (SGLT2)	Sigma-Aldrich	HPA041603		rabbit		1:2000**	1:2000**
SPP1 (OPN)	Santa Cruz	sc-10591		goat		1:100*	
TAGLN (SM22)	Abcam	ab14106		rabbit	1:1000	1:200*	
TGFB1	Abcam	ab215715	EPR21143	rabbit	1:1000		
UQCRFS1	Cell Signaling	95231		rabbit	1:1000		

*The antigens were retrieved by autoclave heating for 5 min in 10 mmol/L citrate buffer (pH 6.0).

**The antigens were retrieved by autoclave heating for 5 min in 1.0 mmol/L ethylenediaminetetraacetic acid buffer (pH 9.0).
